# Changing Molecular Epidemiology of Vibrio cholerae Outbreaks in Shanghai, China

**DOI:** 10.1128/mSystems.00561-19

**Published:** 2019-11-26

**Authors:** Dalong Hu, Zhiqiu Yin, Chao Yuan, Pan Yang, Chengqian Qian, Yi Wei, Si Zhang, Yuhui Wang, Jian Yuan, Meng Wang, Peter R. Reeves, Lihong Tu, Min Chen, Di Huang, Bin Liu

**Affiliations:** a Key Laboratory of Molecular Microbiology and Technology of the Ministry of Education, TEDA College, Nankai University, Tianjin, People’s Republic of China; b TEDA Institute of Biological Sciences and Biotechnology, Nankai University, Tianjin, People’s Republic of China; c Tianjin Key Laboratory of Microbial Functional Genomics, TEDA College, Nankai University, Tianjin, People’s Republic of China; d School of Life and Environmental Sciences, University of Sydney, Sydney, NSW, Australia; e Shanghai Municipal Center for Disease Control and Prevention, Shanghai, People’s Republic of China; f Department of Clinical Laboratory of Tianjin First Central Hospital, Tianjin, People’s Republic of China; Purdue University

**Keywords:** *Vibrio cholerae*, Shanghai cholera, 7th pandemic, whole-genome sequencing, comparative genomics

## Abstract

The 7th cholera pandemic began in 1961 in Sulawesi, Indonesia, and then spread around the world in at least three waves. However, the lack of genome sequences for Vibrio cholerae strains under long-term surveillance in East Asia, especially in China, has restricted our understanding of the dynamics of the intracountry and intercountry evolution and transmission of the 7th-pandemic clones. In this study, we obtained the genome sequences of 60 V. cholerae strains isolated in Shanghai, the largest port in the world and the largest city in China, from 1961 to 2011. Our whole-genome-based phylogeny of 7th-pandemic strains revealed that all but one fell into five “stages,” most of which are single clades and share independent ancestors. Each stage dominated in succession for a period, with little overlap between them. In addition, two near-identical Shanghai strains belonging to a pre-7th-pandemic precursor and 4 nontoxigenic O1/O139 strains attributed to independent recombination events at the O-antigen loci were present. The major lineages of the 7th pandemic in Shanghai appeared to be closely related to V. cholerae strains isolated from South or Southeast Asia. Stage succession was consistently related to changes in society and human activity, implying that human-caused niche change may play a vital role in the cholera dynamics in Shanghai.

**IMPORTANCE** V. cholerae is the causative agent of cholera, a life-threatening disease characterized by severe, watery diarrhea. The 7th pandemic started in Indonesia in 1961 and spread globally, currently infecting 1.3 million to 4 million people annually. Here, we applied whole-genome sequencing to analyze a long-term collection of V. cholerae clinical strains to reveal the phylogenetic background and evolutionary dynamics of the 7th pandemic in Shanghai, which had undergone breathtakingly rapid development in the last half-century. All but one of the Shanghai 7th-pandemic strains fell into five “stages” that were dominant in Shanghai and appeared to be closely related to 7th-pandemic strains of South or Southeast Asia. Our findings extended the understanding of the dynamics of the evolution and transmission of the 7th-pandemic clones in East Asia and the relationship between social changes and cholera epidemiology.

## Abstract (precis)

V. cholerae is the causative agent of cholera, a life-threatening disease characterized by severe, watery diarrhea. The 7th pandemic started in Indonesia in 1961 and spread globally, currently infecting 1.3 million to 4 million people annually. Here, we applied whole-genome sequencing to analyze a long-term collection of V. cholerae clinical strains to reveal the phylogenetic background and evolutionary dynamics of the 7th pandemic in Shanghai, which had undergone breathtakingly rapid development in the last half-century. All but one of the Shanghai 7th-pandemic strains fell into five “stages” that were dominant in Shanghai and appeared to be closely related to 7th-pandemic strains of South or Southeast Asia. Our findings extended the understanding of the dynamics of the evolution and transmission of the 7th-pandemic clones in East Asia and the relationship between social changes and cholera epidemiology.

## INTRODUCTION

Vibrio cholerae is the causative agent of cholera, a life-threatening disease characterized by severe, watery diarrhea (1, 2). There have been seven cholera pandemics since 1817, and all continents except Antarctica have had significant or major incursions by one or more of them (1). The 7th pandemic began in 1961 in Makassar, Sulawesi, Indonesia, and currently causes an estimated 1.3 million to 4 million cases annually (3). An outbreak in Haiti following the 2010 earthquake infected nearly 700,000 people and has caused over 8,500 deaths (4). In a 2008 epidemic in Zimbabwe, more than 90,000 suspected cholera cases were reported, with more than 4,000 patient deaths (5).

The 7th-pandemic El Tor biotype lineage was first observed in the Middle East in 1897 but first caused an outbreak 40 years later (1937) on the Indonesian island of Sulawesi, which was followed by others in 1940, 1944, and 1957 (6, 7). However, after 20 years on Sulawesi, this lineage developed a greater capacity to spread, and a 1960 outbreak in the same location became pandemic in 1961, reaching several countries in Asia, including China, in that year, and then spread around the world in at least three waves. The origins of the 7th pandemic from its appearance in the Middle East to its becoming pandemic in 1961 have been described in a recent paper (8), while the spread of the pandemic strain was covered in an earlier paper (9).

Until very recently, there were very few genome sequences of V. cholerae strains from China. However, Didelot et al. reported a study that included the genome sequences of 71 Chinese isolates and showed that the three major waves in which the 7th pandemic spread around the world all reached China and that one of the strains is related to a prepandemic strain from Indonesia (10). This study provided important insight into the relationship of the strains from the two major centers of cholera, China and South/Southeast Asia.

In this study, we obtained the genome sequences of 60 V. cholerae strains isolated in Shanghai between 1961 and 2011 by the Shanghai Center for Disease Control (CDC). The 60 strains with clear background information, which can be laboratory cultured, were selected as representatives from more than 2,900 isolates in the collection. This is a unique collection of isolates from one city over the entire period of the 7th pandemic. Shanghai is the largest port in the world and the largest city in China; in the past, there have been many outbreaks of cholera, but none of the published genome sequences from China have been reported to be from Shanghai isolates. This study was performed to clarify the evolutionary dynamics of V. cholerae in Shanghai, which had undergone breathtakingly rapid development in the last half-century. Our work may provide novel insights into the relationship between social changes and cholera epidemiology.

In our collection, most of the Shanghai strains were from the 7th pandemic. A phylogenetic analysis was performed for 54 Shanghai 7th-pandemic strain sequences, together with published 7th-pandemic genome data for 146 strains isolated around the world. All but one of the Shanghai 7th-pandemic strains fall into five “stages” that were dominant in Shanghai. Each stage appears to have arrived from South or Southeast Asia. Two Shanghai isolates are related to prepandemic isolates from Indonesia, and four are not related to the major pandemic lineage.

## RESULTS AND DISCUSSION

### Strain choice and analysis based on genome sequencing.

**FIG 1:**
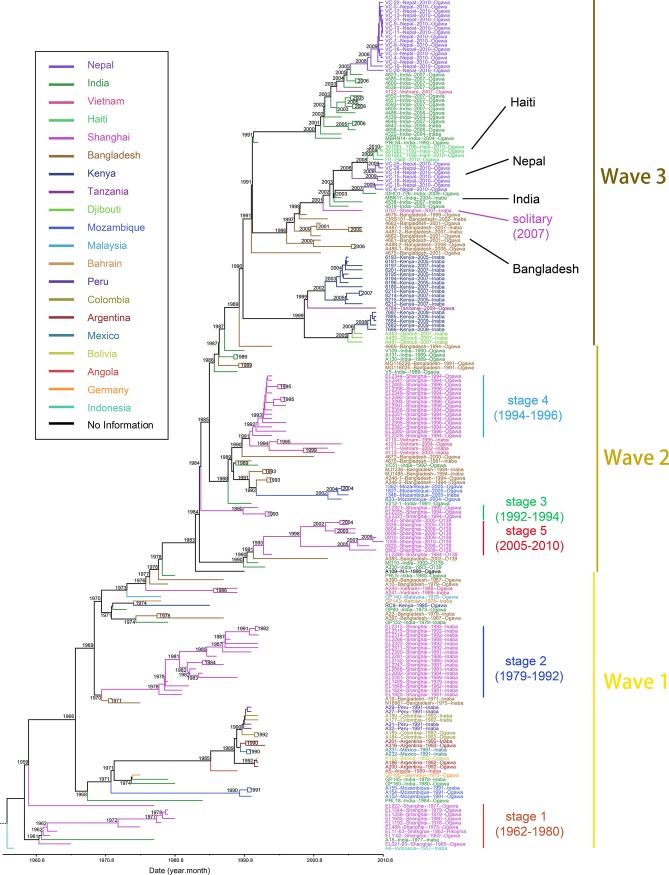
Phylogenetic tree of the 7th-pandemic V. cholerae strains. The maximum likelihood (ML) tree of 200 V. cholerae strains was based on mutational SNPs. The branches of Shanghai isolates corresponding to the five stages in the evolution of the 7th pandemic are labeled with epidemic dates. The dates shown are the estimates for the indicated nodes, taken from the results of the BEAST analysis.

We obtained the genome sequences of 60 V. cholerae strains isolated in Shanghai, which had been retained by the Shanghai CDC and determined to be of the O1 or O139 serogroup and, if O1, the El Tor biotype. We constructed a phylogenetic tree (Fig. 1) based on whole-genome single-nucleotide polymorphisms (SNPs) from our 54 Shanghai 7th-pandemic strains and 146 publicly available 7th-pandemic strains isolated worldwide (see Table S1 in the supplemental material). The SNPs were filtered by a statistical method (11) to identify those introduced by recombination events, and only the recombination event that introduced the O139 O antigen was found. In total, 1,396 of the 1,417 mutational SNPs were congruent with the tree and could be located on specific branches (Fig. S1 and Table S2). Only 21 (1.5%) of the SNPs are not congruent and represent homoplasies involving repeat or reverse events, indicating that the tree structure is very robust. Of the 1,396 congruent SNPs, 1,204 are located in coding regions, of which 846 (70%) are nonsynonymous SNPs (ns SNPs) and 358 are synonymous SNPs. This proportion of nonsynonymous SNPs is very high and indicates positive selection pressure on the 7th-cholera-pandemic strains, but this result could in part be because short-term comparisons are likely to include mutations that will not be retained over longer periods (12).

10.1128/mSystems.00561-19.1 FIG S1 Branch identifications and SNP numbers of the 7th-pandemic phylogenetic tree. Download FIG S1, PDF file, 0.6 MB. Copyright © 2019 Hu et al. 2019 Hu et al. This content is distributed under the terms of the Creative Commons Attribution 4.0 International license.

10.1128/mSystems.00561-19.8 TABLE S1 General characteristics of strains used in this study. Download Table S1, XLS file, 0.06 MB. Copyright © 2019 Hu et al. 2019 Hu et al. This content is distributed under the terms of the Creative Commons Attribution 4.0 International license.

10.1128/mSystems.00561-19.9 TABLE S2 SNPs used to build the 7th-pandemic phylogenetic tree and associated information. Download Table S2, XLS file, 0.3 MB. Copyright © 2019 Hu et al. 2019 Hu et al. This content is distributed under the terms of the Creative Commons Attribution 4.0 International license.

Additionally, for comparison of our strains with the collection reported by Didelot et al. (10), we incorporated their strains into our tree (Fig. S2). We used this tree to place our discussion of the Shanghai strains in the broader context of the whole of China.

10.1128/mSystems.00561-19.2 FIG S2 Phylogenetic tree of our 7th-pandemic collection combined with the China strains from the collection of Didelot et al. (10). Download FIG S2, PDF file, 0.8 MB. Copyright © 2019 Hu et al. 2019 Hu et al. This content is distributed under the terms of the Creative Commons Attribution 4.0 International license.

V. cholerae harbors several key virulence elements, including cholera toxin (CTX), the toxin-coregulated pilus (TCP), *Vibrio* pathogenicity islands (VPIs), and the *Vibrio* seventh-pandemic island (VSP). SXT is an integrative conjugative element that harbors multiple antibiotic resistance genes (13). Genomic islands (GIs) also play an important role in the evolution of V. cholerae, and more than 70 have been observed and defined (14). We also investigated the distribution of these elements (Fig. S3).

10.1128/mSystems.00561-19.3 FIG S3 Virulence-related elements identified among the 216 V. cholerae strains included in this study. The strains are arranged according to the trees shown in Fig. 1 and 3. Download FIG S3, PDF file, 0.7 MB. Copyright © 2019 Hu et al. 2019 Hu et al. This content is distributed under the terms of the Creative Commons Attribution 4.0 International license.

### The five major V. cholerae stages in Shanghai between 1961 and 2011.

The phylogenetic tree in Fig. 1 shows that 54 of the 7th-pandemic Shanghai isolates fall into five stages, most of which are single clades that dominated the isolates in succession. Each stage shares an independent ancestor and appears to have arrived independently. The five major stages in Shanghai are shown in Fig. 1. Four of these are in clades reported by Didelot and Wilson (15) (Fig. S3). The features and affiliations of the isolates from the five stages are discussed below. The changes for genetic elements in each stage and the SNPs specific to each stage are also discussed below.

### Stage 1 (1962 to 1980).

Stage 1 includes 7 O1 Ogawa strains and one (EL11-63) O1 Hikojima strain (Fig. 1). All have the CTX pathogenicity island and the El Tor RS1, toxin-linked cryptic (TLC), and VPI-1 elements but do not have the self-transmissible, chromosomally integrating genetic (SXT) element drug resistance island (Fig. S3). Most of the stage 1 strains fit within the China 1.A clade described by Didelot et al., and in both collections, the earliest strain was isolated in 1962, and the last strain was isolated in 1978 or 1980. The locations of the isolates described by Didelot et al. were not reported.

**FIG 2:**
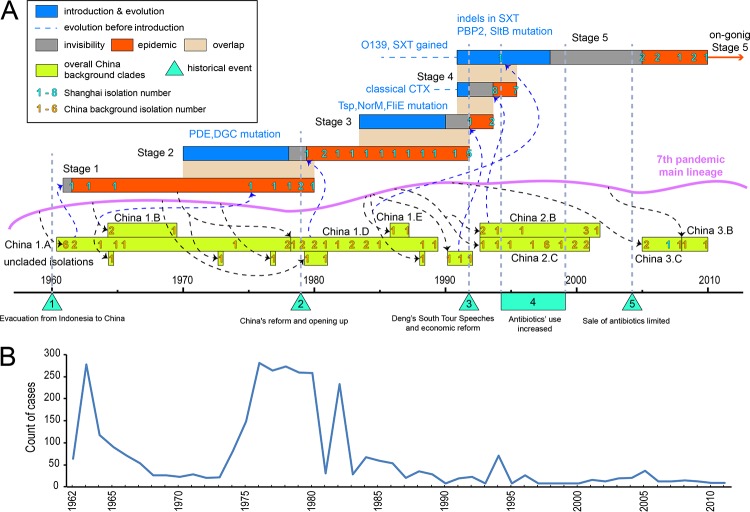
Details of the 5 stages of the Shanghai cholera outbreak. (A) Date ranges of each evolutionary and transmission process of the five stages, shown as color-coded horizontal bars, with details of the mutational events and sequenced isolate numbers given for each stage. The important historical events in Shanghai and/or China are also labeled. The correspondence of Shanghai isolates to the China background isolates and the introduction of China clades from the 7th-pandemic main lineage are linked by blue and black dotted arrows, respectively. (B) Line chart showing the numbers of cholera cases in Shanghai from 1962 to 2011.

The 7th pandemic began in 1961 and was also first reported in China in 1961 (16). Our first Shanghai isolate is from 1962, and its clade is located at the root of the 7th-pandemic phylogenetic tree. This rapid transfer to China may be related to the migration of many Chinese people from Indonesia to China at that time (16) (Fig. 2A). The last stage 1 strain was isolated in 1980 (Fig. 1 and Fig. 2A), at a time when isolates from South and Southeast Asia were all from later-diverging branches of the tree. According to the case report (Fig. 2B), the outbreaks in the early 1960s and from 1975 to 1980 in Shanghai correspond well to the stage division.

All strains but EL922 share a common ancestor on branch 381 (Fig. S1) and are part of the stage 1 clade, while EL922 arrived independently (Fig. 1). Only one ns SNP on the *rho* gene (Table S2), which encodes a transcription termination factor (17), is located on branch 381 leading to stage 1. There is no ns SNP on branch 381. That indicates that these strains might already have been able to cause outbreaks in Shanghai as they arrived.

### Stage 2 (1979 to 1992).

The stage 2 strains have the same GI patterns as those of the stage 1 strains (Fig. S3) but are O1 Inaba strains with the same *rfbT* mutation as N16961, which is the root of the stage 2 clade (18). The Shanghai stage 2 strains fall within the China group, China clade 1.D (Fig. S2).

The first isolate is from 1979, just before the last stage 1 isolate from 1980. Transfer occurred on branch 299 (Fig. S1), which diverges very close to the branch to a well-characterized Bangladesh strain, N16961. Two ns SNPs on branch 299 (Table S1) are in pathogenicity-related genes, including genes for a c-di-GMP phosphodiesterase A-related protein (PDE) and a GGDEF family protein (diguanylate cyclases [DGC]) (Fig. S4). The PDE and DGC genes participate in the synthesis and decomposition of c-di-GMP, which is a second messenger generally found in bacteria and can regulate many physiological processes, such as the generation of virulence factors, motility, and the promotion of biomembrane synthesis (19, 20).

10.1128/mSystems.00561-19.4 FIG S4 Mutations in stages 2, 3, and 5. Download FIG S4, PDF file, 1.1 MB. Copyright © 2019 Hu et al. 2019 Hu et al. This content is distributed under the terms of the Creative Commons Attribution 4.0 International license.

**FIG 3:**
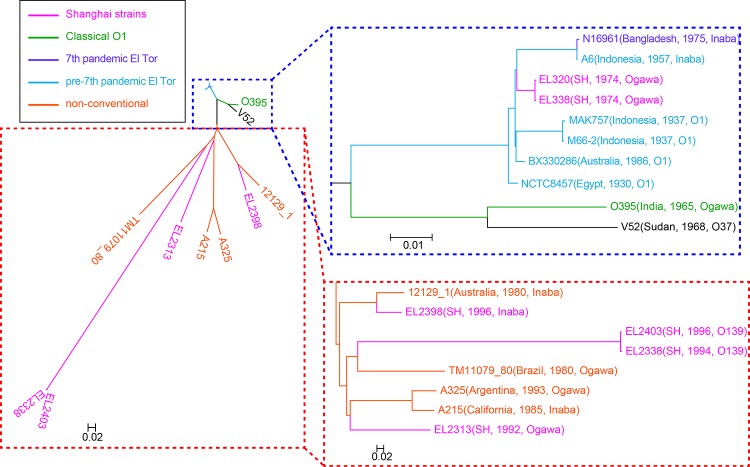
Phylogenetic tree for 6th-pandemic and nonconventional strains combined with pre-7th- and 7th-pandemic strains. SH, Shanghai.

The replacement of stage 1 strains with stage 2 strains occurred between 1978 and 1980. During that time, China started its greatest recent political and economic reform, called “China’s reform and opening up” (Fig. 3). From 1978 to 1980, many special economic zones were established, and private enterprise, especially in the import and export trades, was opened. We suggest that these great changes in the social environment could have driven the succession of cholera in Shanghai during this period, although the connection between the genetic variations and the socioeconomic changes is unclear due to the complexity of China’s reform and opening up, which covered every aspect of China’s society. Future studies are required to confirm the function of those mutations and their correlation with socioeconomic changes.

### Stage 3 (1992 to 1994).

There are only 3 Shanghai strains in the stage 3 lineage that are connected to branch 251 (Fig. 1). They are O1 Ogawa strains, as in stage 1, and the GI pattern is the same as that of stages 1 and 2 (Fig. S3). Three strains reported by Didelot et al. are clustered in stage 3, as shown in Fig. S2, but they were not given a clade name. We suggest that this shared group be named China clade 1.F (Fig. S2).

There are 21 mutations on branch 251 (Fig. S1) dating from 1984 to 1990, and some of them are in genes that might be related to bacterial drug resistance, survival, and motility (Fig. S4). They include (i) a gene for a tail-specific protease (TSP), which is involved in the cleavage of a C-terminal peptide of 11 residues from the precursor form of penicillin-binding protein 3 (PBP3) and may be involved in bacterial defense against thermal and osmotic stresses (21); (ii) a gene encoding the multidrug resistance protein NorM, which is a multidrug efflux pump that functions as a Na^+^/drug antiporter and confers resistance to several drugs, including norfloxacin, ciprofloxacin, ethidium, kanamycin, and streptomycin (21); and (iii) the gene for the flagellar hook-basal body complex protein FliE, which is located in the flagellar basal body and functions as a structural adaptor for flagellin secretion (22, 23). The number and nature of the mutations on branch 251 suggest that clade 3 underwent adaptive evolution before its first isolation in 1992 (Fig. 3).

Similar to the stage 2 lineage, stage 3 strains also underwent a relatively long local evolution process before their first isolation in 1992. Then, a special economic zone was established in Shanghai, which may have caused a second abrupt environmental change. These environmental changes may have caused a lineage with better selective advantages to become the dominant population. The end of the prevalence of stage 3 strains may be related to a change in the use of antibiotics. In approximately 1994, the use of cephalosporin started to increase dramatically (24).

### Stage 4 (1994 to 1996).

Stage 4 strains are O1 Ogawa strains, similar to stage 1, but their GI content differs from those of stages 1 to 3 in the replacement of the El Tor CTX island with the classical CTX island, as observed for the parental lineage (Fig. 1 and Fig. 2A). Stage 4 strains fall within China clade 2.B described by Didelot et al. (Fig. S2), when there was an outbreak in Shanghai in 1994 (Fig. 2B).

The introduction to China occurred on branch 194, which has no mutations, but there is one SNP on branch 195, which leads to all isolates but EL2329. The mutation is in the *lepA* gene, which encodes a GTP-binding protein. Branch 194 diverges close to the branch for a group of Vietnam strains (Fig. 1 and Fig. S1). The classical CTX island of stage 4 strains may have been acquired by their South Asian ancestor in 1988 on branch 190 (Fig. 1, Fig. 2A, and Fig. S1).

### Stage 5 (2005 to 2010).

Stage 5 strains are of serogroup O139 and have the same GI pattern as those of the stage 1 to 3 strains, except for the presence of the drug resistance island SXT (Fig. 1 and Fig. S3). The O139 variant of the 7th pandemic was first isolated in India in 1992 and soon came to dominate in South and Southeast Asia. From 1978 to 1984, this branch gained the SXT island (9). However, after 1995, the number of clinical O139 cases decreased (25). In contrast, nearly all of the clinical V. cholerae cases reported in Shanghai after 2005 were of serogroup O139 (Fig. 1 and Fig. 2A). A similar phenomenon has been observed for the prevalence of O139 isolates in Mainland China (26). The O139 lineage reached Shanghai on branch 259 (Fig. S1), which was estimated to span from 1991 to 1993, soon after it was first reported in India, and the first isolate reported in Shanghai was from 1994. However, it did not become dominant until 2005 (Fig. 2A), perhaps due to the increase in antibiotic use between 1994 and 1999 (24). O139 strains were not present in the set described by Didelot et al., but as the latest isolate in that collection was from 2005, this lack does not mean that this serogroup was not widespread in China generally during the period of its presence in Shanghai.

The 3 mutations on branch 259 are all synonymous. However, there are several mutations affecting proteins that may influence virulence on subsequent branches. The mutations on branch 260 represented the genetic changes between EL2388 (in 1994) and other O139 strains (2005 to 2010), which may reveal key evolutionary dynamics during this period. All strains except for EL2388 (Table S1) carry two nonsynonymous mutations in the well-known drug-related genes *pbp2*, which encodes penicillin-binding protein 2 (PBP2) (27), and *sltB*, which encodes a lytic murein transglycosylase, with a function in beta-lactam antibiotic resistance (28) (Fig. S4). These mutations are on branch 260, which was estimated to run from 1993 to 1998 (Fig. 1 and Fig. S1), when the use of beta-lactam antibiotics increased dramatically in Shanghai. O139 isolates from other areas do not have mutations in these two genes. These genetic changes may explain how the stage 5 O139 lineage became dominant in Shanghai, and the increment of antibiotic resistance of these stage 5 strains has also been confirmed by antimicrobial susceptibility experiments (Table S3).

10.1128/mSystems.00561-19.10 TABLE S3 Antimicrobial susceptibility of strains isolated in Shanghai. Download Table S3, XLS file, 0.02 MB. Copyright © 2019 Hu et al. 2019 Hu et al. This content is distributed under the terms of the Creative Commons Attribution 4.0 International license.

The SXT islands of strains EL2388 and 0902 are the same as or similar to that of the early O139 strain MO10. However, many stage 5 strains, such as strains 0910, 1009, and 0802, have experienced different deletion events, most of which are related to the loss of trimethoprim resistance (Fig. S5). The lack of trimethoprim resistance in these strains must be a result of convergent evolution. Trimethoprim resistance may have had a weak contribution to the survival of O139 strains in the Shanghai environment at that time (24).

10.1128/mSystems.00561-19.5 FIG S5 Genetic organization of the SXT regions of stage 5 O139 strains. Download FIG S5, PDF file, 0.2 MB. Copyright © 2019 Hu et al. 2019 Hu et al. This content is distributed under the terms of the Creative Commons Attribution 4.0 International license.

### Summary of the 7th pandemic in China and Shanghai.

In general, good concordance exists between the strains in the two collections. The major exception, the absence of clade 2.C isolates in Shanghai, is probably due to a lack of samples from 1997 to 2004 in the Shanghai collection. However, the absence of stage 5 O139 isolates in the rest of China may reflect a real difference in Shanghai. However, the existing data include relatively few strains for such a large country, so it is not possible to say how much the O139 strain actually spread in China.

### Solitary strain 0707.

The 2007 0707 O1 Inaba strain is the only wave 3 Shanghai isolate (Fig. 1). It has the “hybrid CTX,” El Tor RS1, TLC, VPI-1, and SXT GI pattern, with some deletion events as well as some other GIs (Fig. S3). It is related to the Bangladesh CIRS1010 strain from a lineage of South Asian strains, which is prevalent in Nepal and was recently transmitted from there to Haiti to cause the Haiti cholera outbreak in 2010 (29).

### Two isolates of prepandemic strains related to the Makassar outbreaks.

Two nearly identical 1974 O1 Ogawa clinical isolates (EL320 and EL338) (Fig. 3) diverged from the main pathogenic lineage about midway between the divergence of the 1937 prepandemic outbreak in Indonesia and the divergence of the 1957 prepandemic outbreak in Indonesia from the main lineage leading to the 7th pandemic (8).

These two strains do not have RS1 elements (Fig. S3), and their CTX island is a hybrid type (with the classical *ctxB* gene and the El Tor *rstR* gene, while the CTX of wave 3 strains have the El Tor *ctxB* gene and the classical *rstR* gene [30]) that has never been found in other V. cholerae strains. The variation in CTX from 6th-pandemic strains to the recent 7th-pandemic strains is displayed in Fig. S6. The CTX types of the two Shanghai strains fill the gap in the evolution of El Tor CTX. From the end of the 6th pandemic to the recent 7th pandemic, the CTX island, which is a key virulence element of V. cholerae, evolved from the classical type to the El Tor type and then back to the classical type, which indicates that V. cholerae can adapt to environmental changes by changing its virulence.

10.1128/mSystems.00561-19.6 FIG S6 Evolution of the CTX type. Download FIG S6, PDF file, 0.4 MB. Copyright © 2019 Hu et al. 2019 Hu et al. This content is distributed under the terms of the Creative Commons Attribution 4.0 International license.

### Four nontoxigenic O1/O139 strains are the result of independent recombination events at the O-antigen loci.

Four of the Shanghai clinical isolates are nontoxigenic and branch from the known pathogenic lineage well before the divergence of the classical and El Tor pandemic lineages (Fig. 3). They do not have the CTX, RS1, TLC, and SXT GIs or even VPI-1, which carries the *tcp* gene cluster (Fig. S3). Thus, it is concluded that the O1 and O139 antigen phenotypes arose by horizontal gene transfer, respectively, in the evolution of these nontoxigenic strains, in agreement with data from a previous study (9). Except for the *wbfA* gene, the major region of the O-antigen gene cluster of nonconventional O139 strains shares high identity (near the average identity of housekeeping genes between these two lineages) to that of the typical 7th-pandemic O139 strains, similar to the O-antigen gene cluster of the O22 serogroup, for which the evolution process is thought to be caused mainly by recombination events (Fig. S7). The finding that these strains are from cholera cases can be attributed to independent recombination events at the O-antigen loci, including the O139 antigen.

10.1128/mSystems.00561-19.7 FIG S7 Alignment of O139 O-antigen gene clusters of Shanghai nonconventional strains with those of related lineages. Download FIG S7, PDF file, 0.7 MB. Copyright © 2019 Hu et al. 2019 Hu et al. This content is distributed under the terms of the Creative Commons Attribution 4.0 International license.

### Conclusions.

We found that 54 of the 60 7th-pandemic strains isolated in Shanghai between 1962 and 2010 cluster into five clones. Each stage was dominant for a period, with very little overlap between periods of dominance, although the small number of isolates available would not show the presence of a clone at lower numbers outside its period of dominance. This situation is very different from the situation in South and Southeast Asia, where there was much greater diversity during that period, as shown by a previous report (9). It is striking that the South/Southeast Asian lineage was the source of each of the five stages of the samples that we observed. This result was quite unexpected, as historically, South/Southeast Asia and China were the two areas in which cholera had been reported for centuries, but it appears that for the 7th pandemic, at least, South and Southeast Asia were the center of evolution for cholera. Additionally, the transitions between each stage were reliably associated with social changes in China, especially Shanghai, showing the importance of human social activities in the dynamic evolution of the Shanghai 7th pandemic.

## MATERIALS AND METHODS

### Strains and culture conditions.

All strains used in this are listed in Table S1 in the supplemental material. The 60 V. cholerae strains (listed in Table S1) were kindly provided by the Shanghai Municipal Center for Disease Control and Prevention. All strains were isolated from clinical specimens and stored at −80°C in Luria-Bertani (LB) broth with 20% glycerol. LB broth at 37°C was used as the standard bacterial growth condition.

### Genome sequencing.

DNA was prepared from 1 ml of cultures grown overnight with the Wizard genomic DNA purification kit (Promega) according to the manufacturer’s instructions. We obtained draft genome sequences for 60 V. cholerae strains (Table S1) using Solexa pair-end sequencing (Illumina, Little Chesterford, Essex, United Kingdom), with a depth of 90- to 100-fold coverage, and the sequences were *de novo* assembled using Velvet v1.0 (31). Protein prediction and annotation of the newly sequenced genomes were performed by using Glimmer v3.0 (32) and BLASTp v2.6.0^+^ (33), using the 1 October 2018 version of the NCBI nr database (34), respectively. The annotation result was verified manually using the annotation of the genome sequence of reference strain N16961.

### SNP calling and recombination detection.

The SNPs across the genome of each strain were identified by aligning and mapping against the reference genome sequence of strain N16961 using Mauve v2.3.1 (35) and BWA v0.7.13 (36). We used RecDect v1.0 within SaRTree pipeline v1.1 (11) to analyze and remove SNPs due to recombination.

### Phylogenetic analyses and divergence time analysis.

The maximum likelihood (ML) phylogenetic tree was built by using RAxML v8.2.11 (37) with 1,000 bootstraps and the –m GTRGAMMER parameter, including 1,417 mutational SNPs (Table S2). These mutational SNPs were grouped into different patterns according to the strains in which they occur, and these patterns were then allocated to specific branches of the ML tree by using SaRTree pipeline v1.1.

We used BEAST v1.10.4 (38) to infer the divergences dates of the lineages. The BEAST estimations were performed using the evolutionary model GTR+Γ4 (general time reversible model with gamma distributions) with log-normal relaxed molecular clocks, the coalescent constant population size, and 10,000,000 Markov chain Monte Carlo (MCMC) states sampled every 1,000 steps. Different models and parameters were tested by parallel runs of BEAST, and the best model used in this study was selected by comparing the marginal likelihoods, when all the effective sample sizes (ESSs) were >200 for all the parameters, using Tracer v1.5. The first 5% of trees were then discarded as burn-ins, and the posterior probability limit filtering threshold was set to 50%. The divergence dates were estimated using the remaining trees, which were mapped onto the maximum likelihood phylogeny generated in the last step with the mean height of the nodes using TreeAnnotator v1.10.4. The output tree was displayed by using FigTree v1.4.4.

### Comparative genomics.

To examine virulence elements and GI-1 to GI-67, we located and screened genes or gene clusters using BLASTp v2.6.0^+^ (33). The reference sequences of GIs were determined as described in a previous article (14). Protein secondary structure was predicted using PSIPRED v4.0 (39).

### Antimicrobial susceptibility.

An antimicrobial susceptibility experiment was performed for the Shanghai strains using the Etest (AB Biodisk, Solna, Sweden), according to the manufacturer’s instructions, on LB agar plates, with incubation at 37°C for 18 to 24 h.

### Data availability.

Sequence data are available in the NCBI RefSeq database under project accession no. PRJNA555013. The accession numbers of the 60 strains’ genomes are listed in Table S1 in the supplemental material.
