# Supplementary figures and images for: Changing Molecular Epidemiology of Vibrio cholerae Outbreaks in Shanghai, China
Source: mSystems. 2019 Nov 26;4(6):e00561-19. doi: 10.1128/mSystems.00561-19 (PMC6880041; doi:10.1128/mSystems.00561-19)

(Number of SNPs, Number of Recombination regions)

~~[Branch No.]~~

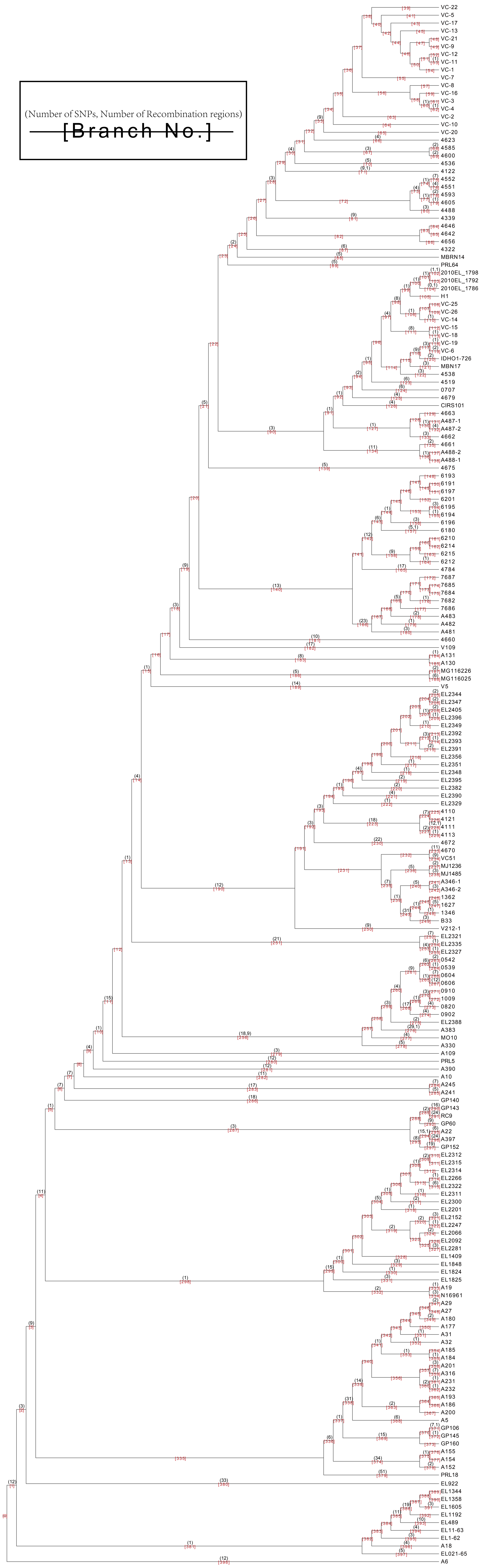

Supplement: FIG S1 [file mSystems.00561-19-sf001.pdf]

PDE

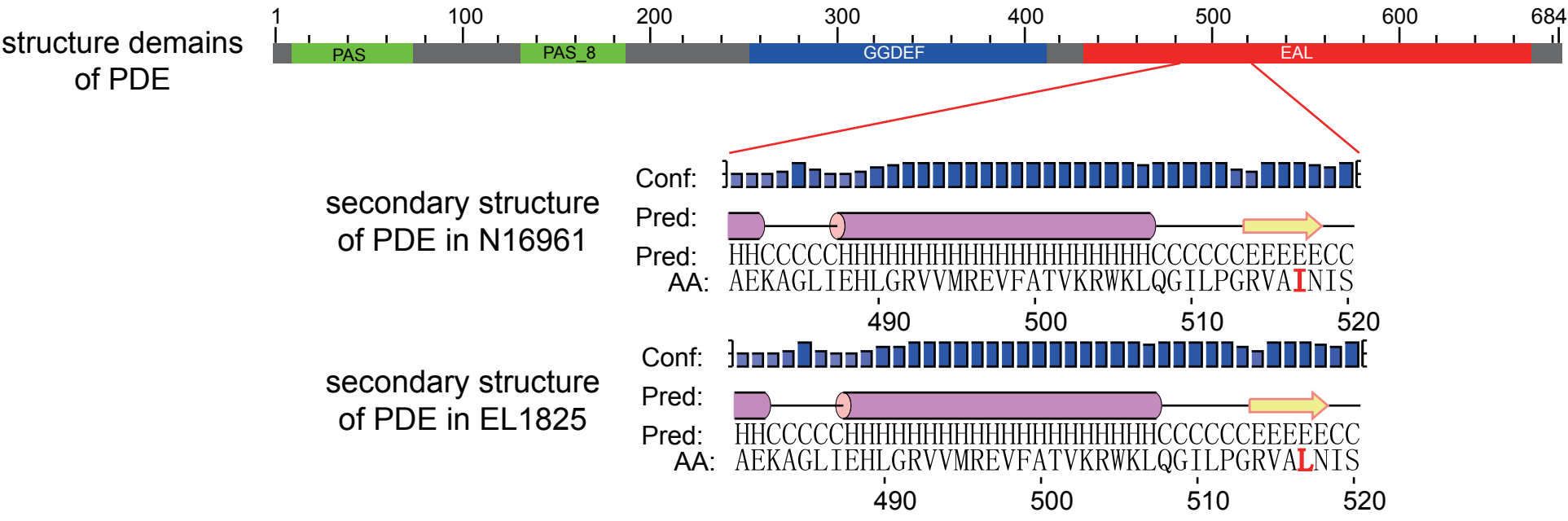

DGC

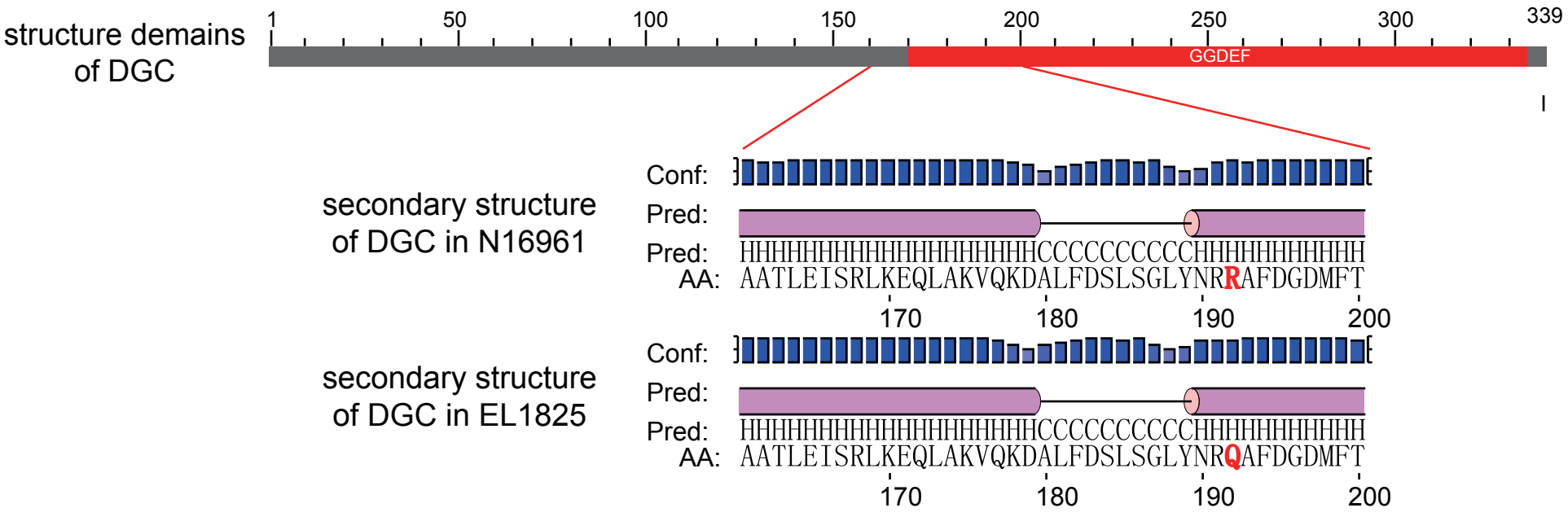

TSP

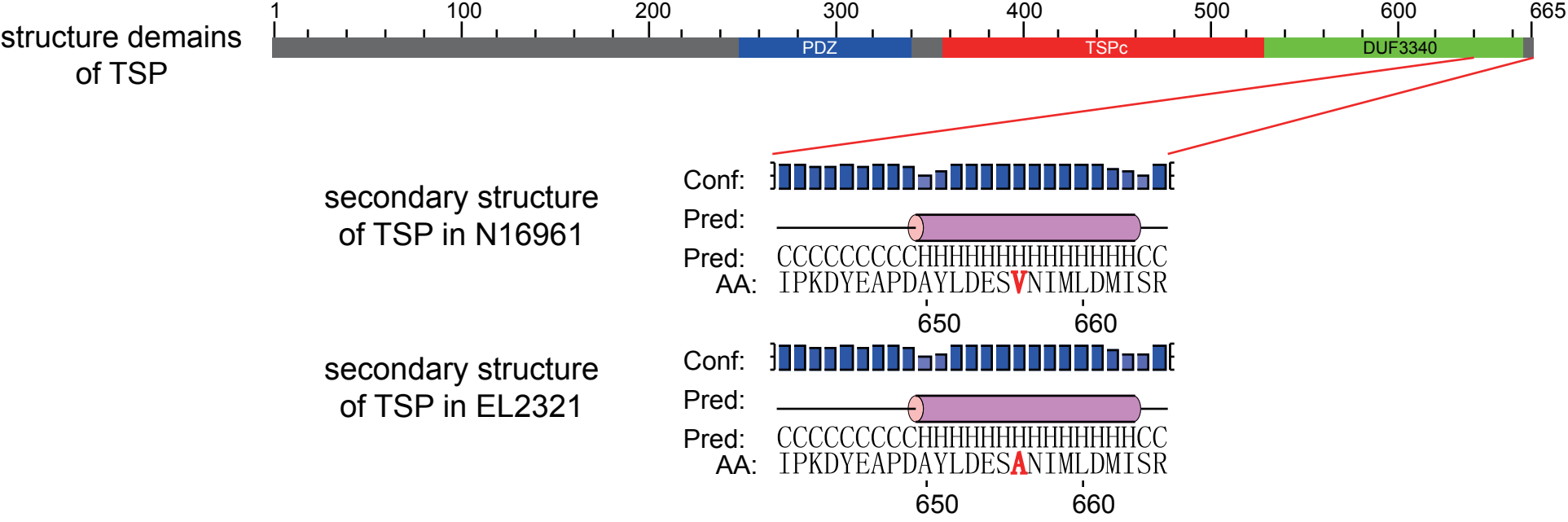

NorM

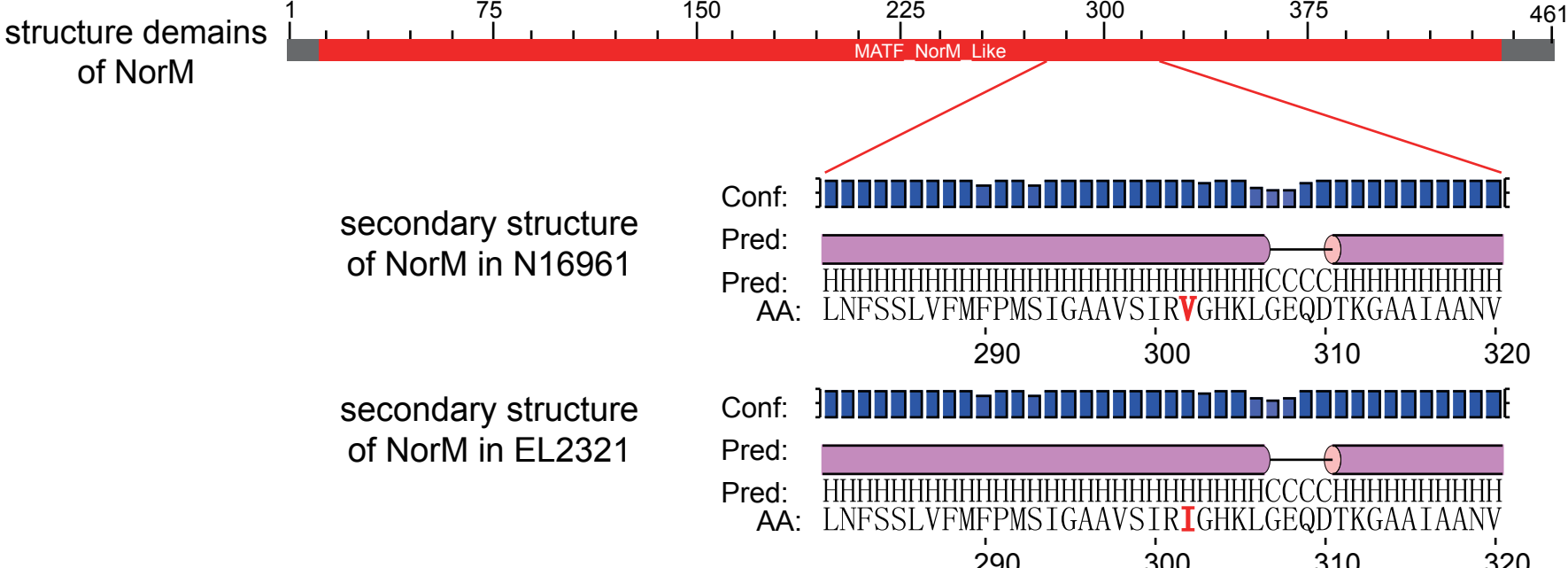

File

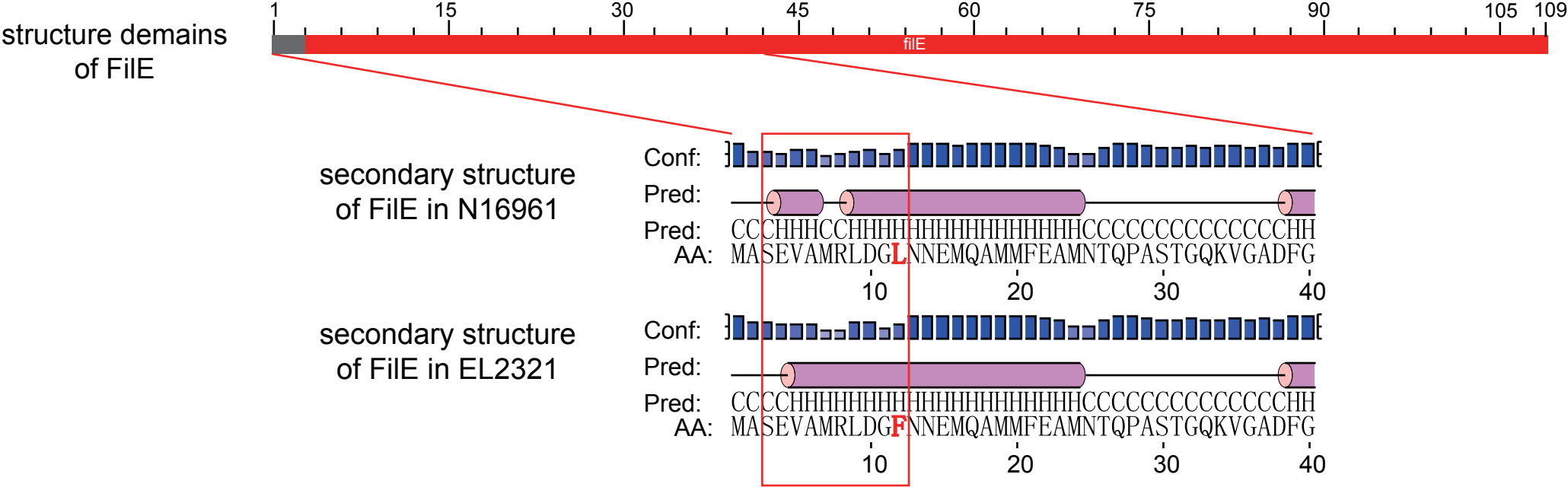

PBP2

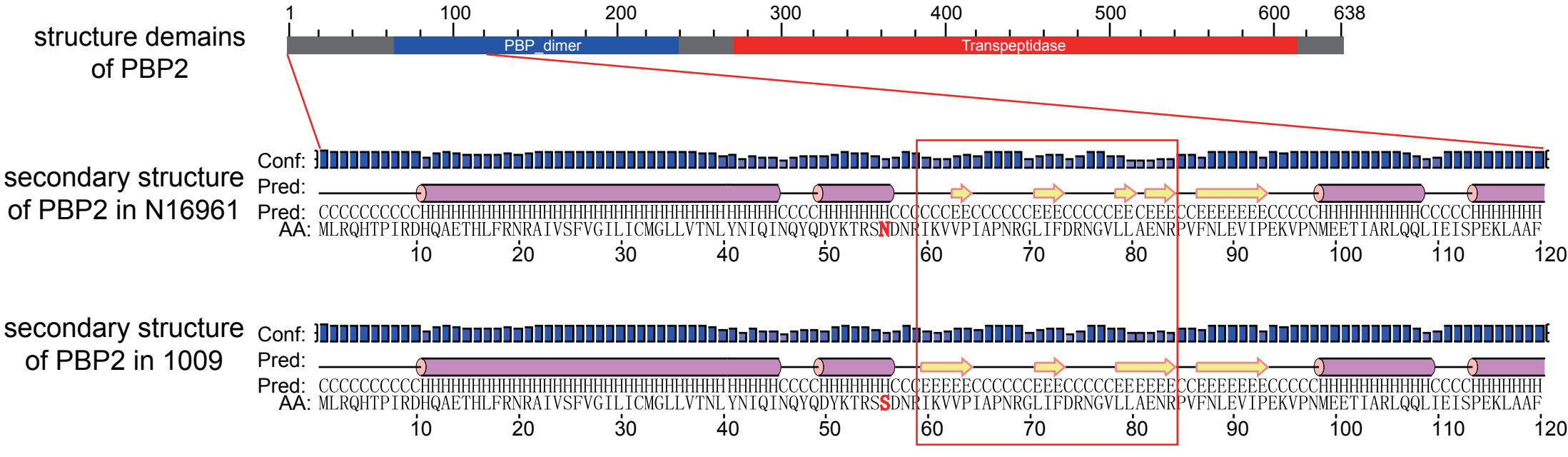

SlkB

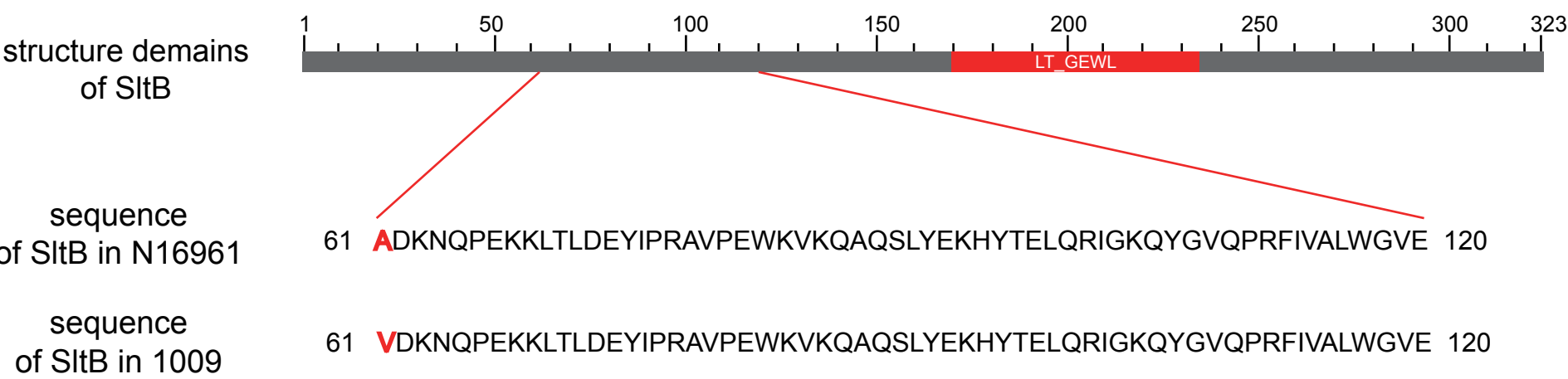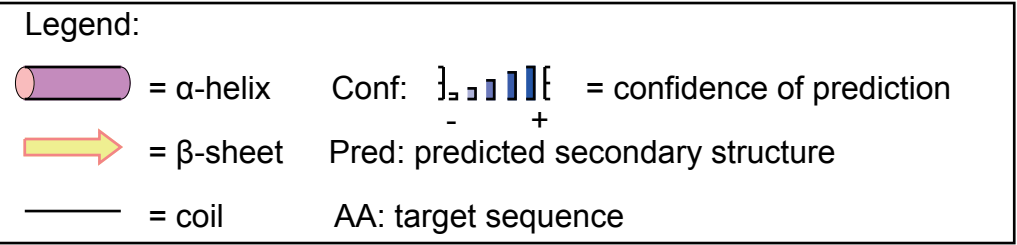

Supplement: FIG S4 [file mSystems.00561-19-sf004.pdf]

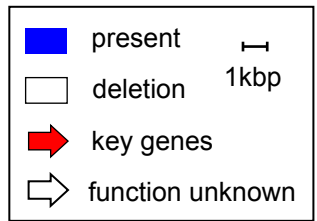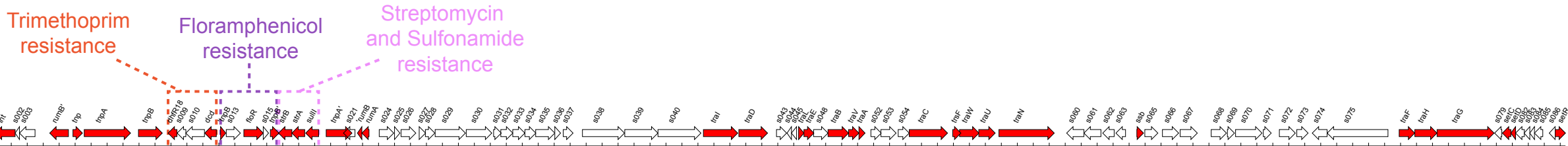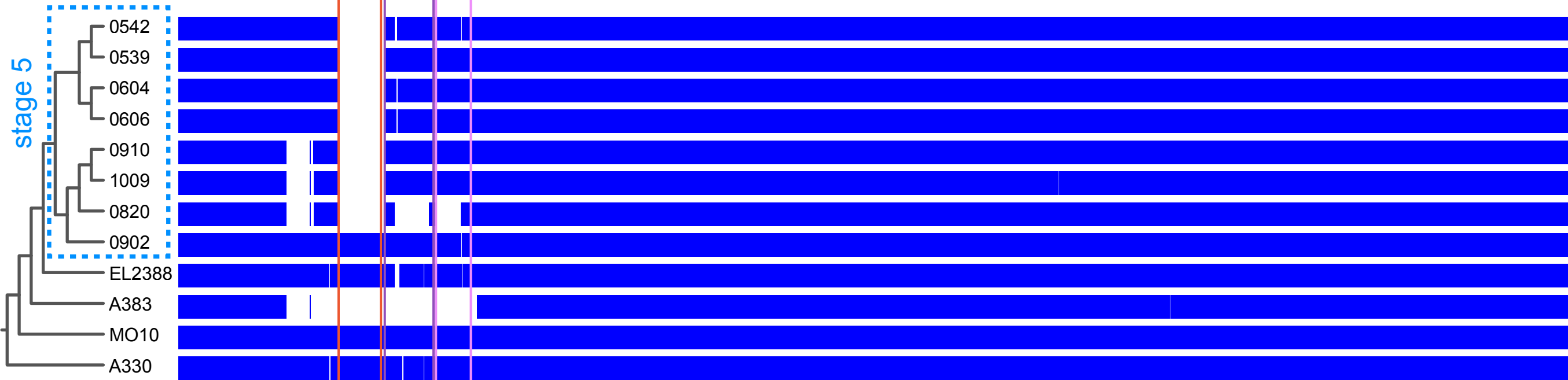

Supplement: FIG S5 [file mSystems.00561-19-sf005.pdf]

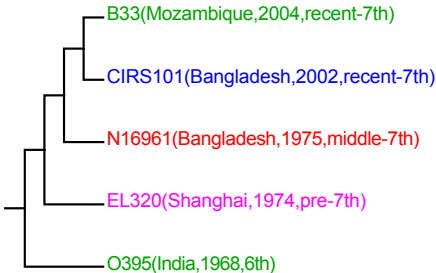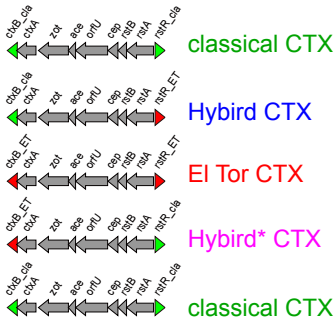

Supplement: FIG S6 [file mSystems.00561-19-sf006.pdf]
