# Supplementary material for: Changing Molecular Epidemiology of Vibrio cholerae Outbreaks in Shanghai, China
Source: mSystems. 2019 Nov 26;4(6):e00561-19. doi: 10.1128/mSystems.00561-19 (PMC6880041; doi:10.1128/mSystems.00561-19)
Supplement: FIG S2 [file mSystems.00561-19-sf002.pdf]

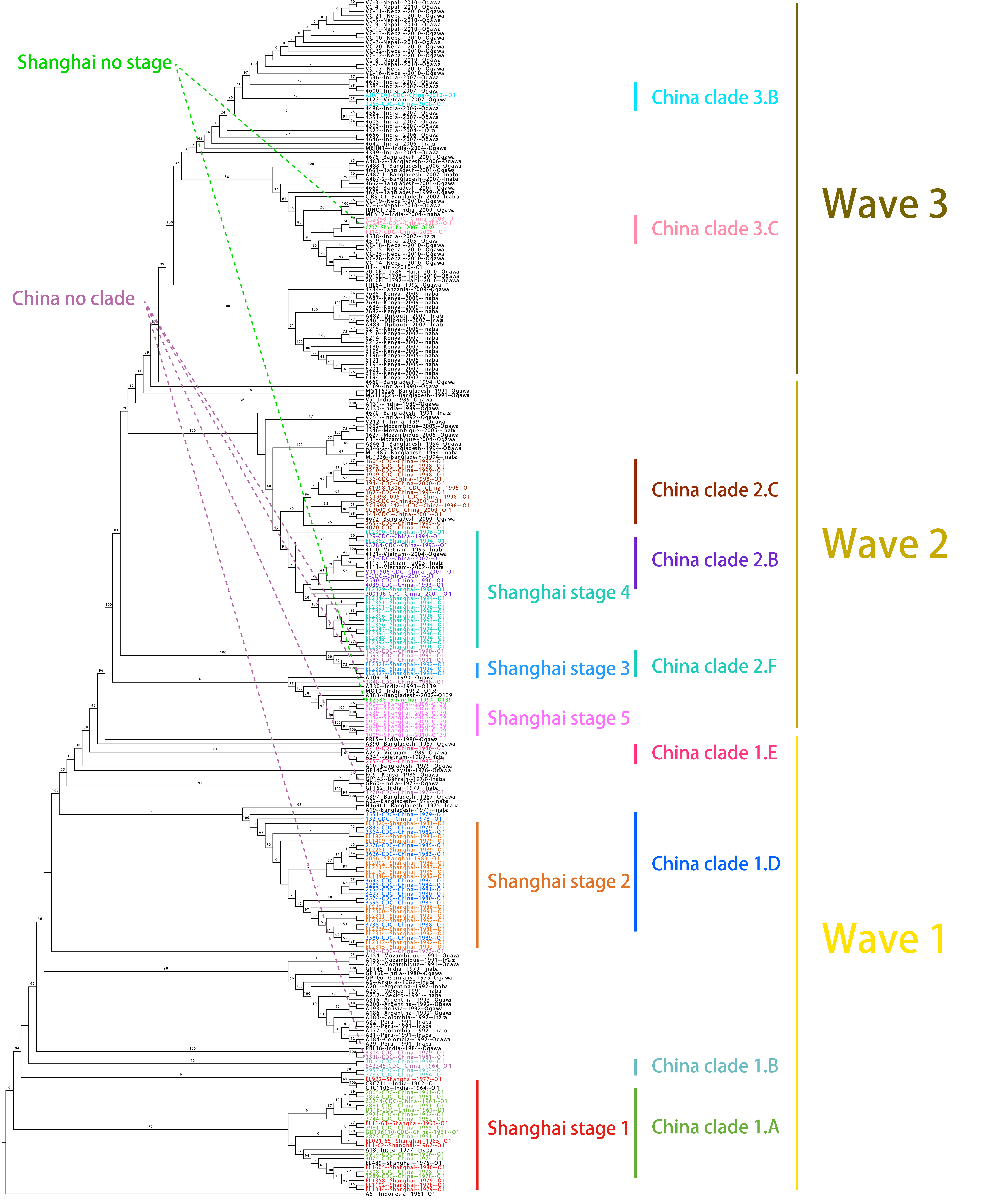

Shanghai no stage
China no clade
China clade 3.B
China clade 3.C
Wave 3
China clade 2.C
China clade 2.B
Shanghai stage 4
Wave 2
Shanghai stage 3
China clade 2.F
Shanghai stage 5
China clade 1.E
China clade 1.D
Shanghai stage 2
Wave 1
China clade 1.B
Shanghai stage 1
China clade 1.A
