# Supplementary material for: Changing Molecular Epidemiology of Vibrio cholerae Outbreaks in Shanghai, China
Source: mSystems. 2019 Nov 26;4(6):e00561-19. doi: 10.1128/mSystems.00561-19 (PMC6880041; doi:10.1128/mSystems.00561-19)
Supplement: FIG S3 [file mSystems.00561-19-sf003.pdf]

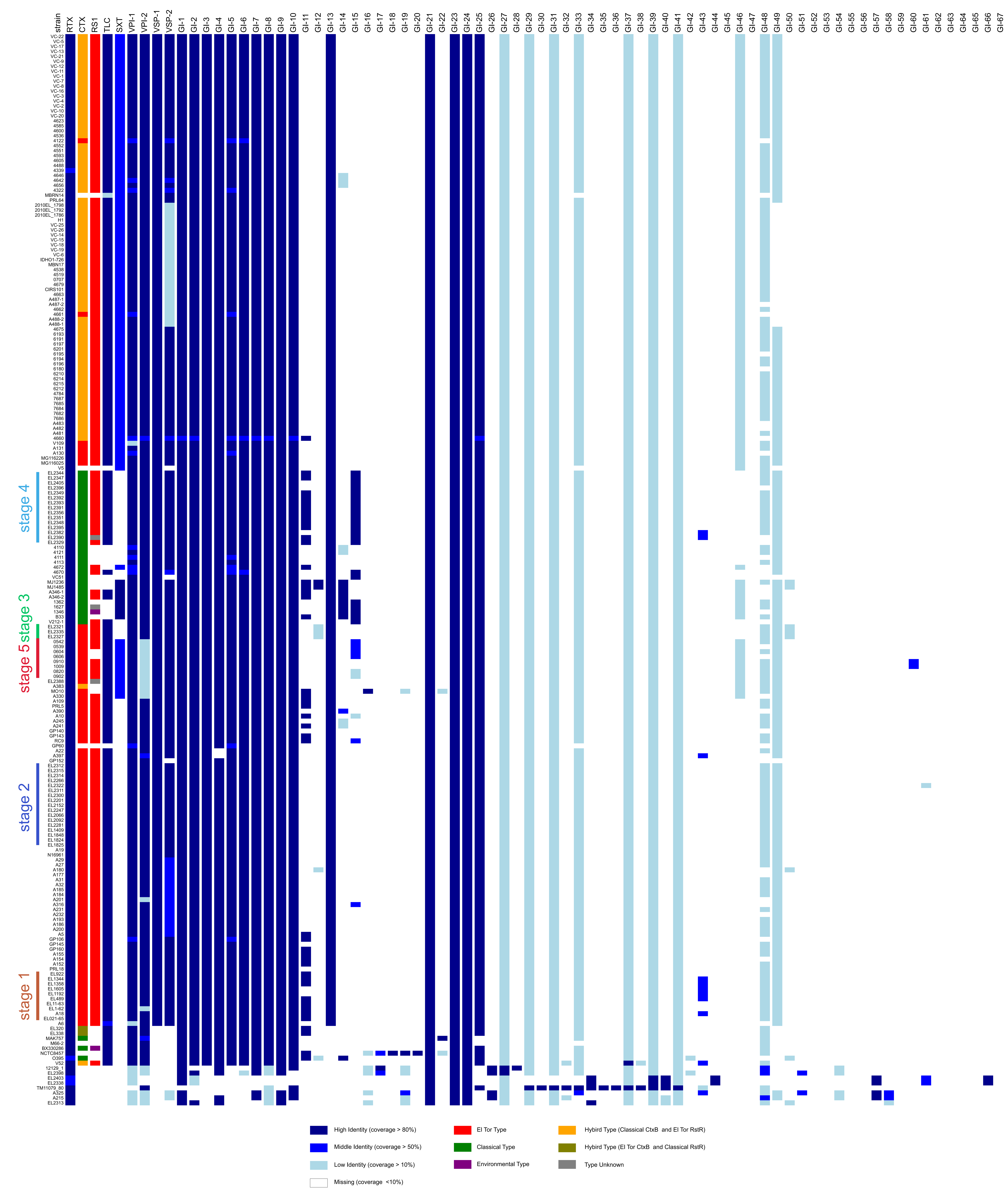
strain
RTX
CTX
RS1
TLC
SXT
VPI-1
VPI-2
VSP-1
VSP-2
GI-1 … GI-67
stage 4
stage 3
stage 5
stage 2
stage 1
High Identity (coverage > 80%)
Middle Identity (coverage > 50%)
Low Identity (coverage > 10%)
Missing (coverage <10%)
El Tor Type
Classical Type
Environmental Type
Hybird Type (Classical CtxB and El Tor RstR)
Hybird Type (El Tor CtxB and Classical RstR)
Type Unknown
