# Supplementary material for: Changing Molecular Epidemiology of Vibrio cholerae Outbreaks in Shanghai, China
Source: mSystems. 2019 Nov 26;4(6):e00561-19. doi: 10.1128/mSystems.00561-19 (PMC6880041; doi:10.1128/mSystems.00561-19)
Supplement: FIG S7 [file mSystems.00561-19-sf007.pdf]

O-antigen gene cluster

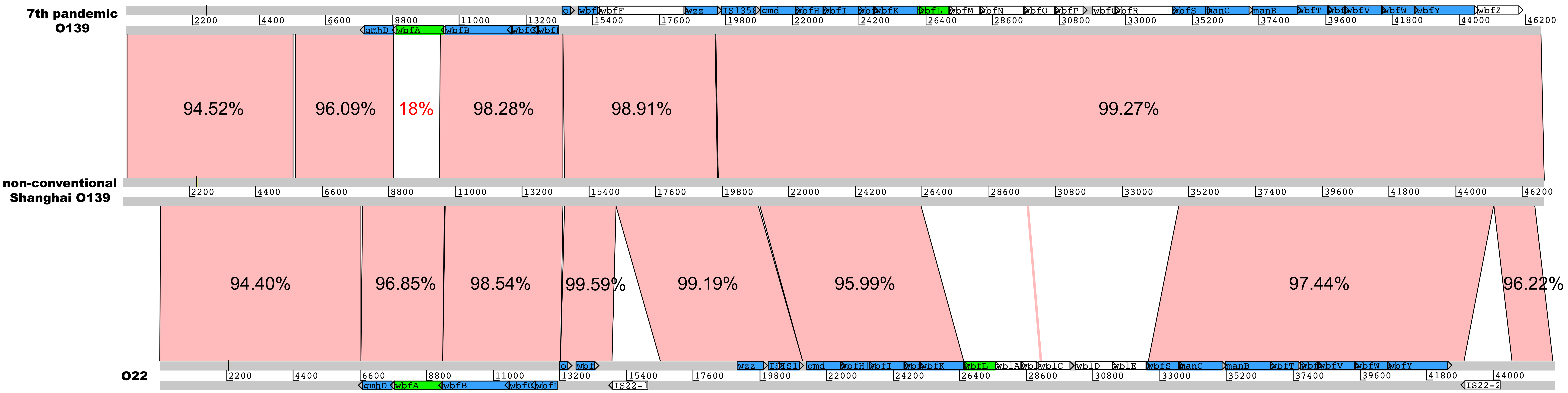

7 Housekeeping genes in N16961	adk	gyrB	metE	mdh	pntA	purM	pyrC
Identity to non-conventional Shanghai O139 strains	98%	94%	95%	96%	97%	99%	91%
Identity to 7th pandemic O139 strains	100%	100%	100%	100%	100%	100%	100%
